# Intrahematomal Ultrasound Enhances RtPA-Fibrinolysis in a Porcine Model of Intracerebral Hemorrhage

**DOI:** 10.3390/jcm10040563

**Published:** 2021-02-03

**Authors:** Julia Masomi-Bornwasser, Axel Heimann, Christian Schneider, Tristan Klodt, Hammoud Elmehdawi, Andrea Kronfeld, Harald Krenzlin, Yasemin Tanyildizi, Karl-Friedrich Kreitner, Oliver Kempski, Clemens Sommer, Florian Ringel, Naureen Keric

**Affiliations:** 1 Department of Neurosurgery, University Medical Center of the Johannes Gutenberg University, 55131 Mainz, Germany; scchrist@students.uni-mainz.de (C.S.); Harald.Krenzlin@unimedizin-mainz.de (H.K.); florian.ringel@unimedizin-mainz.de (F.R.); naureen.keric@unimedizin-mainz.de (N.K.); 2 Institute for Neurosurgical Pathophysiology, University Medical Center of the Johannes Gutenberg University, 55131 Mainz, Germany; axel.heimann@unimedizin-mainz.de (A.H.); oliver.kempski@unimedizin-mainz.de (O.K.); 3 Institute of Neuropathology, University Medical Center of the Johannes Gutenberg University, 55131 Mainz, Germany; tristan.klodt@unimedizin-mainz.de (T.K.); clemens.sommer@unimedizin-mainz.de (C.S.); 4 Department of Neuroradiology, University Medical Center of the Johannes Gutenberg University, 55131 Mainz, Germany; Elmehdawi@gmx.de (H.E.); Andrea.Kronfeld@uni-mainz.de (A.K.); yasemintanyildizi@googlemail.com (Y.T.); 5 Department of Diagnostic and Interventional Radiology, University Medical Center of the Johannes Gutenberg University, 55131 Mainz, Germany; karl-friedrich.kreitner@unimedizin-mainz.de

**Keywords:** intracerebral hemorrhage, ultrasound-thrombolysis, rtPA-fibrinolysis, minimally invasive therapy

## Abstract

Catheter-based ultrasound-thrombolysis has been successfully used in a small clinical trial in order to enhance recombinant tissue plasminogen activator (rtPA)-fibrinolysis, for the treatment of spontaneous intracerebral hemorrhages (ICHs). The aim of this study was to investigate the ultra-early effects of ultrasound on hematoma and the surrounding brain tissue in a porcine ICH-model. To achieve this, 21 pigs with a right frontal ICH were randomly assigned to four groups: (1) drainage (*n* = 3), (2) drainage + rtPA (*n* = 6), (3) drainage + ultrasound (*n* = 6), and (4) drainage + ultrasound + rtPA (*n* = 6). The hematoma volume assessment was performed using cranial MRI before and after the treatments. Subsequently, the brain sections were analyzed using HE-staining and immunohistochemistry. The combined treatment using rtPA and ultrasound led to a significantly higher hematoma reduction (62 ± 5%) compared to the other groups (Group 1: 2 ± 1%; Group 2: 30 ± 12%; Group 3: 18 ± 8% (*p* < 0.0001)). In all groups, the MRI revealed an increase in diffusion restriction but neither hyper- or hypoperfusion, nor perihematomal edema. HE stains showed perihematomal microhemorrhages were equally distributed in each group, while edema was more pronounced within the control group. Immunohistochemistry did not reveal any ultra-early side effects. The combined therapy of drainage, rtPA and ultrasound is a safe and effective technique for hematoma-reduction and protection of the perihematomal tissue in regard to ultra-early effects.

## 1. Introduction

The treatment of spontaneous, intracerebral hemorrhage (ICH) is still subject to debate, and guidelines concerning evidence-based, first-line therapy and the role of surgical treatment are needed in the future [1]. There are believed to be 5 million incidences of ICH per year worldwide [2]. This fatal subtype of stroke has the highest rate of death, and a one-year mortality of approximately 50%. After 6 months, 61–80% of survivors are still dependent on help in daily life [3]. Craniotomy studies have failed to show any improvement in survival and functional outcome in ICH patients [4,5] (with the exception of superficial lobar ICHs) [6].

In a meta-analysis of 15 high-quality randomized controlled trials (with a 3–12 month follow up), minimally invasive surgery (MIS) for ICH (including endoscopic surgery and stereotactic thrombolysis by rtPA), increased the chance of being independent by 2.2 times and the survival by 1.7 times, when compared to conventional craniotomy and conservative treatment (performed within 24–72 h) [7]. It is difficult to draw conclusions about any subgroup superiority in these wide-scattered studies, as the heterogeneity of influencing factors like age, follow-up time, hematoma volume and location, study size, scope of collected data, time until treatment, inflammation, oxidative stress, toxicity of blood degradation products and scope of monitoring, all have to be considered.

Recently, the MISTIE III trial failed to show an improvement in clinical outcome after the treatment of supratentorial ICHs with a volume of 30 mL or greater via the intralesional application of 1 mg/mL rtPA through a stereotactically inserted catheter [8]. Nevertheless, there was an increase in survival without an increase in severe disability, seemingly associated with a reduction in ICH volume [8]. This modest effect has to be treated with caution, as it is based on a secondary analysis; however, there is evidence from multiple sources that ICH volume reduction is a major influencing factor of MIS [8,9,10].

One implication of the MISTIE II trial is that an ICH-volume below 15 mL after therapy has to be reduced in more patients in the future [8]. An enhancement of hematoma fibrinolysis using rtPA by catheter-based intrahematomal ultrasound application (2 MHz) was performed in a clinical study of nine patients [11]. Compared with the MISTIE and CLEAR data, it has been observed that a faster rate of lysis occurred in the patients treated with sonothrombolysis plus rtPA compared to rtPA alone during treatment for ICH [11].

In the last two decades, a similar approach has been propagated for the enhancement of intravenous rtPA fibrinolysis of intraarterial clots in ischemic stroke therapy via the application of transcranial ultrasound [12,13,14,15,16,17,18,19,20,21,22]. However, the potential of ultrasound thrombolysis can cause side effects such as heat development, brain edema and ICH [13,14,17,18,20].

Side effects of high rtPA doses [23,24,25] have been reported, such as edema and ineffective further fibrinolysis through the faster consumption of the rtPA binding partner plasminogen [26].

In our in vitro clot model of ICH, we have previously shown that 1 mg rtPA with an exposure time of 15 min is the lowest possible optimal dose and timing [27]. We also systematically examined the enhancement of the intrahematomal rtPA application by a 10 MHz high-frequency ultrasound catheter, placed inside the clot. Clot fibrinolysis was significantly enhanced (40% compared to rtPA alone), with a radius suitable for the size of an ICH, without any side effects such as heating [28].

The aim of the following study is to examine the effectiveness of the combined treatment of ICH with rtPA and intrahematomal ultrasound in order to evaluate possible side effects to the surrounding brain tissue in an established porcine ICH model [29].

## 2. Materials and Methods

### 2.1. Animals and Surgical Preparation

Our study was approved by the Animal Welfare Agency of Rhineland Palatinate (Landesuntersuchungsamt Koblenz) in accordance with the current guidelines of the Animal Welfare Act and with the institutional guidelines for animal welfare and experimental conduct of the University Medical Centre of the Johannes Gutenberg University Mainz. We confirm that the paper follows the rules of the Declaration of Helsinki and that the experiments have been conducted according the AARRIVE Guidelines.

An established porcine model of ICH was used for this study and a sample size of six per group was chosen according to the literature (Wagner et al., 1996), whereas three animals were chosen for the control group, where a reduced variance of parameters was assumed [29]. Twenty-one Deutsche Landrasse male pigs between 30 and 35 kg were sedated using a mixture of midazolam (0.3–0.5 mg/kg), azaperone (2 mg/kg) and atropine (0.033 mg/kg). A venous line was placed in the ear or leg. Thiopental (7–10 mg/kg) and piritramide (7.5 mg) were used for intubation. A femoral arterial and venous lines were placed. In order to maintain narcosis, thiopental (125–200 mg/h) and piritramide (10–15 mg/h) were applied via the femoral venous line. The animals were mechanically ventilated using a respirator (Servo Ventilator 900 B^®^, Siemens Healthcare, Erlangen, Germany), which was adjusted in order to achieve physiological limits of pH (7.4), arterial blood gases and expiratory carbon dioxide (CO2) (6.3–6.5 kPa) (spirometry monitoring: Capnomac Ultima^®^, Datex, Maintal, Germany). Blood gas analysis was performed every 3 h. A volume-controlled ventilation was chosen using a tidal volume of 100–125 mL/kg, a flow of 4.2 ± 1.5 L/min and a breathing frequency of 19–25/min, with a fraction of inspired oxygen (FiO2) of 25–26%. A rectal thermometer measured core temperature, which was maintained at a constant, physiological level by an electric homeothermic blanket. Intra-arterial blood pressure, heart rate and electrocardiography (ECG) were continuously monitored throughout the experiments. This procedure took approximately 2–2.5 h.

### 2.2. ICH Preparation

**Figure 1:**
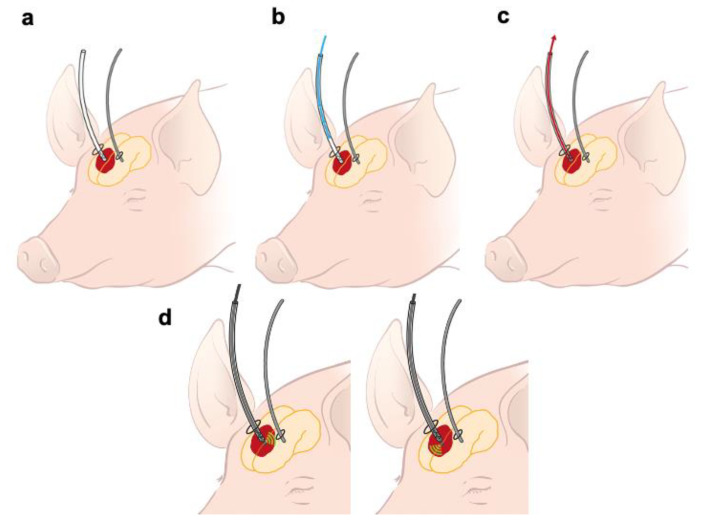
(**a**) This image shows a schematic illustration of a pig with an intrahematomal (red) catheter (white) in the right frontal area and an intracranial pressure (ICP) probe (grey) in the left frontal area; (**b**) Lysis-catheter inside the hematoma, the blue arrow illustrates the application of 1 mg rtPA after the initial MRI. (**c**) After treatment, the intrahematomal catheter was connected to a gravity-based drainage system for one hour (red arrow). Afterwards, an MRI of the brain was repeated. (**d**) Intrahematomal catheter after the first MRI. Inside the lumen of the lysis-catheter there is the ultrasound-catheter (black), absorbing ultrasound, cone-shaped laterally (green waves) for 30 min in each direction. After this time, it is turned 180° to the other direction (right image).

After shaving and disinfection, a midline incision was made. A left frontal burr hole (0.5 cm) was placed for continuous intracranial pressure (ICP) measurement via an ICP probe (Raumedic, Helmbrechts, Germany) (Figure 1a). A second burr hole (1 cm diameter) was placed 1cm right of the sagittal suture and 2.3 cm anterior of the coronal suture, through which a Fogarty catheter (Edwards, Irvine, CA, USA) was placed, 2.5 cm below the bone surface inside the right frontal lobe. The catheter was slowly inflated with 5 mL of air for a time period of 2 min, after which the air was slowly deflated and removed. Afterwards, 5 mL of arterial autologous blood was taken from the femoral arterial line. This was infused into the prepared ICH cavity via a 10 F catheter, through the right frontal burr hole (1 cm). The infusion was kept at a constant rate of 3 mL/min by a pressure-controlled infusion pump (TSE Systems, Ratshausen, Germany). ICP was measured during this procedure. After the ICH procedure, the tip of a shortened catheter 16 CH (ConvaTec, Munich, Germany) was placed into the ICH and orientated orthogonally 2.5 cm from bone. The catheter had 4 holes of 4 mm diameter, laterally of the catheter, at the last 2 cm of the tip (Figure 1a). The catheter was closed immediately after positioning.

### 2.3. Magnetic Resonance Imaging (MRI)

After ICH, all pigs (still under sedation), underwent a 3 Tesla-MRI (MAGNETOM Prisma^®^, Siemens Healthcare, Erlangen, Germany) examination using a 4-channel flexible coil. This covered: T1- and T2-weighted structural imaging, diffusion-weighted imaging (DTI), non-contrast agent, and contrast agent-based perfusion imaging.

Structural imaging consisted of T1-, T2*-, T2-, and proton density (PD)-weighted protocols. In addition, a fluid attenuated inversion recovery (FLAIR) protocol was used to suppress any signal from fluids close to the affected tissue. T1-weighted imaging was used, with a magnetization prepared rapid gradient echo (MPRAGE), and an isotropic voxel size of 0.7 mm. A fast low-angle shot (FLASH) pulse sequence was used to obtain T2*-weighted images, with an in-plane resolution of 0.7 mm × 0.7 mm, and a slice thickness of 3 mm.

T2- and PD-contrast were achieved by a dual echo turbo spin pulse sequence, with an in-plane resolution of 0.56 mm × 0.56 mm, and a slice thickness of 3 mm. FLAIR images were acquired by a turbo inversion magnitude (TIRM) sequence and a spatial resolution equal to the T2-/PD-weighted images.

The diffusion weighted data were collected by echo planar imaging (EPI) with an in-plane resolution of 1.4 mm × 1.4 mm and a slice thickness of 3 mm. Apparent diffusion coefficient (ADC), fractional anisotropy (FA) and trace-weighted images were calculated inline by the scanner software.

Perfusion data were obtained by the non-contrast agent-based technique: arterial spin labeling (ASL), as well as by a contrast agent (CA)-based susceptibility weighted technique. With the ASL-technique, an in-plane resolution of 2.8 mm × 2.8 mm and a slice thickness of 6 mm was achieved. Relative blood flow and perfusion-weighted maps were calculated inline by the scanner software. A susceptibility-weighted measurement was then started, based on an EPI-pulse sequence. After 10 s of baseline measurements, a contrast agent (Gadovist^®^, Bayer Vital GmbH, Leverkusen, Germany, 0.1 mL/kg BW) was applied manually through the femoral venous catheter during the ongoing measurement. Images were acquired with an in-plane resolution of 1.4 mm × 1.4 mm and a slice thickness of 3 mm. Perfusion parameters were calculated by the scanner software.

In order to evaluate hematoma volume, the intersection volume of T2* and T1 was measured. Edema was defined as the volume of hyperintensity in the FLAIR images without the diffusion restriction volume in the diffusion images. The volume of hyperintensity in DTI images was measured in order to evaluate diffusion restriction. To measure the volume of edema, diffusion restriction and surrounding bleeding, the iPlan^®^ software (Brainlab, Munich, Germany) was used as a volumetric tool for ICH volume and the Sectra^®^ software (Sectra Medical, Linköping, Sweden). The cranial MRI was repeated after therapy and analyzed in the same manner.

### 2.4. ICH Treatment-Groups

All animals were randomly assigned into four treatment groups (Figure 1): Group 1 (drainage only; *n* = 3), Group 2 (drainage + rtPA (1 mg); *n* = 6), Group 3 (drainage + ultrasound catheter; *n* = 6), and Group 4 (drainage + ultrasound catheter + rtPA; *n* = 6).

Group 1 (drainage only; *n* = 3): After MRI, the intrahematomal catheter was connected to a gravity-based drainage system (Neuromedex), which was placed 10 cm below the auditory canal of the pigs and drained for 1 h (Figure 1a,c). To prevent the cessation of drainage because of high blood viscosity, the drainage system was flushed with sterile 0.9% saline solution in a distal direction.

Group 2 (drainage + rtPA (1 mg); *n* = 6): In this group, 1 mg/mL rtPA (Actilyse^®^, Boehringer Ingelheim, Ingelheim, Germany) followed by 1 mL of sterile 0.9% saline solution was administered via the intralesional catheter to avoid remnants of rtPA inside the dead volume of the catheter. An optimal incubation of 15 min, which was described by Keric et al., 2017, was followed by a drainage period of one hour (Figure 1a–c).

Group 3 (drainage + ultrasound catheter; *n* = 6): In this group, the 10 F ACUNAV™ endosonography catheter (Biosense Webster, New Brunswick, NJ, USA) was placed inside the ICH through the intralesional catheter. This catheter absorbs ultrasound by its cone shaped 22° × 90° field of view laterally, so one side of the ICH was insonated for 30 min. Then, the catheter was turned by 180°. An insonation of 30 min of the other side of the ICH was performed. This optimal insonation time was chosen as previously found in our in vitro experiments [28]. The ACUSON SEQUOIA™ 512 ULTRASOUND SYSTEMS (Siemens Healthcare, Erlangen, Germany) was used for ultrasonic treatment and imaging, which was adjusted to the lowest possible mechanical index (MI) of 0.55. After that, the liquefied part of the ICH was drained for 1 h in the same way as all other groups (Figure 1a,c,d).

Group 4 (drainage + ultrasound catheter + rtPA; *n* = 6): As for group 2, 1 mg of rtPA was administered inside the ICH, and rtPA was flushed out of the catheter into the ICH, using 1 mL of sterile 0.9% saline solution. Additionally, the endosonography catheter was placed inside the mixture of ICH and rtPA, and insonation was performed for one hour, as described for Group 3. This was followed by a drainage period of one hour (Figure 1a–d).

Cranial MRI was performed after the completion of all treatments. Four animals had to be excluded, and one animal of Group 4 died prior to ICH induction because of cardiovascular breakdown. Technical problems occurred during the MRI so that imaging was enabled and experiments were delayed in an unacceptable and not comparable manner, which affected one animal of Group 2 and one of Group 3. Technical problems and catheter dislocation during the transport were experienced in one animal of Group 2.

### 2.5. Fixation and Sampling

All pigs remained in deep anesthesia, and after an intravenous bolus of Thiopental, euthanasia was performed by the intravenous administration of potassium chloride 6 h after ictus. The brains were fixed using 4% paraformaldehyde and embedded in paraffin. Slices were made next to the ICH (area 1), contralaterally frontal to the ICH (area 2), and posterior ipsi- (area 3), and contralaterally (area 4) to the ICH.

### 2.6. Staining and Immunohistochemistry

Hematoxylin and eosin (HE) staining was used to semi-quantify microhemorrhages and edema in areas 1 to 4. Visualization of immediate early neuronal, astroglial, and microglial activation, was performed by the c-fos protein (Fos), glial fibrillary acidic protein (GFAP), and the acting binding protein Iba1. A severity score ranging from 1 to 3 was developed-. 1 = no activation, 2 = mild activation and 3 = strong activation.

### 2.7. Statistical Analysis

Statistical analyses and graphs were created using GraphPad PRISM^®^ (version 8) (GraphPad Software, Inc., La Jolla, USA). One-way analysis of variance was used for comparative analysis. Two-sided *p*-values below 0.05 were considered statistically significant. The Tukey-Kramer method was applied for all pairwise post-hoc comparisons and report adjusted *p*-values. The Spearman’s rank-correlation coefficient was used to assess the correlation between diffusion restriction before and after treatment, and the ICH volume before and after treatment. For all parameters, 95% confidence intervals and means ± standard deviations (SD) were reported.

## 3. Results

### 3.1. Hematoma Volume

**Figure 2:**
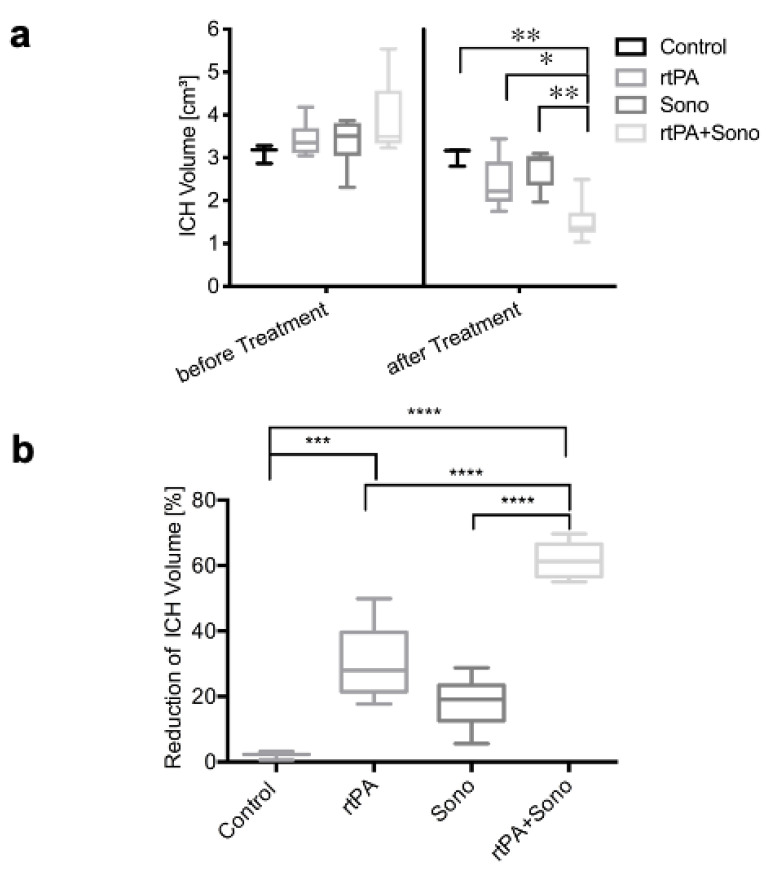
(**a**) Hematoma volumes (*y*-axis) [cm³] are illustrated as box plots before and after treatments (*x*-axis). ** marks the significant volume difference after treatment of the rtPA- and sono-treated group and the control group of *p* = 0.002 and between the ultrasound-treated group and the combined treatment group of *p* = 0.0023. * marks the significant volume difference after therapy between the group with the combined treatment and the group treated with rtPA of *p* = 0.0278. (**b)** Relative hematoma volume reduction after treatment (*y*-axis) [%] of the different groups (*x*-axis) is shown using box plots. **** marks the significant hematoma volume reduction in group 4 of *p* < 0.0001. *** marks the significant volume reduction in group 2 compared to group 1 of *p* = 0.0007.

Before treatment, all groups showed a similar volume of ICH (mean ± SD): The control Group (*n* = 3) showed a volume of 3.11 ± 0.22 cm³, Group 2 (rtPA) (*n* = 6) had a volume of 3.44 ± 0.42 cm³, Group 3 (ultrasound) (*n* = 6) showed a volume of 3.38 ± 0.56 cm³ and Group 4 (rtPA+ ultrasound catheter) (*n* = 6) had a volume of 3.89 ± 0.88 cm³ (Figure 2a). The combined treatment using ultrasound and rtPA led to the lowest hematoma volume of 1.51 ± 0.5 cm³, which was significantly lower than the volume of the control group 3.05 ± 0.21 cm³ (*p* = 0.002) the volume of the group treated with rtPA 2.41 ± 0.61 cm³ (*p* = 0.0278) and the volume of Group 3 (ultrasound) 2.75 ± 0.44 cm³ (*p* = 0.0023) (Figure 2a).

The combined treatment led to a volume reduction of 62 ± 5%, which was significantly higher than the volume reduction in Group 3 (ultrasound): 18 ± 8% and group 2 (rtPA): 30 ± 12% and the control Group 2 ± 1% (*p* < 0.0001) (Figure 2b). The volume reduction in Group 2 was also significant compared to the control Group (*p* = 0.0007).

### 3.2. Vital Signs

**Figure A1:**
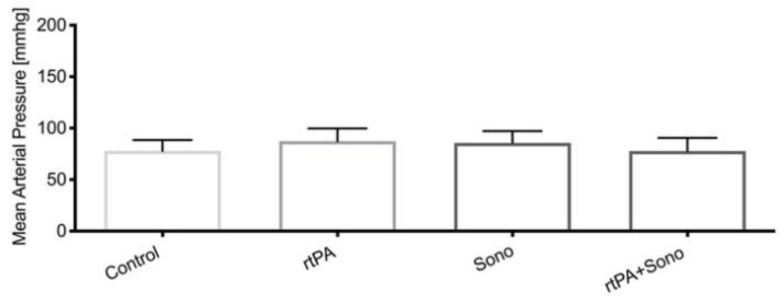
Mean arterial pressure (MAP) of the groups (control Group *n* = 3; treatment groups (2–4); each *n* = 6). The means and SD of the MAP are plotted as a column bar graph.

**Figure A2:**
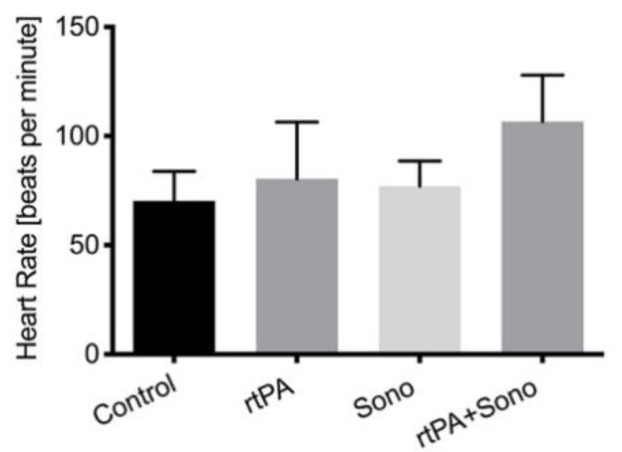
Heart rate (HR) of the groups (control Group *n* = 3; treatment groups (2–4); each *n* = 6). The means and SD of the HR are plotted as a column bar graph.

**Figure A3:**
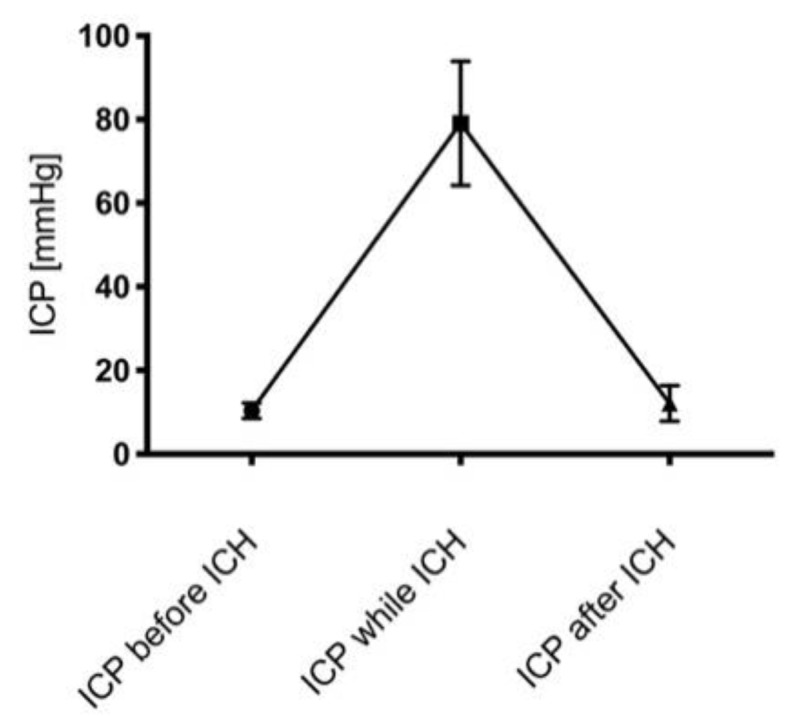
Intracranial pressure (ICP) (control Group *n* = 3; treatment groups (2–4); each *n* = 6). The means and SD of the ICP before, while and after ICH induction are plotted.

Animals from all groups showed comparable vital signs (mean ± SD). The mean arterial pressure (MAP) of the control group was 77.93 ± 10.42 mmHg, the MAP of Group 2 was 87.42 ± 12.28 mmHg, Group 3 showed a MAP of 85.66 ± 11.43 mmHg and Group 4 had a MAP of 77.79 ± 12.71 mmHg (Appendix A, Figure A1). The mean heart rate (HR) of the control group was 70.33 ± 13.44 beats per minute (bpm), the mean HR of Group 2 was 80.48 ± 25.9 bpm, group 3 showed a mean HR of 77.03 ± 11.58 bpm and Group 4 had a mean HR of 106.7 ± 21.25 bpm (Appendix A, Figure A2). The mean ICP of all groups was 10.36 ± 1.83 mm Hg before ICH, 79.13 ± 14.85 mm Hg, while ICH placement and 12.15 ± 4.24 mm Hg after ICH (Appendix A, Figure A3).

### 3.3. Cranial MRI

**Figure 3:**
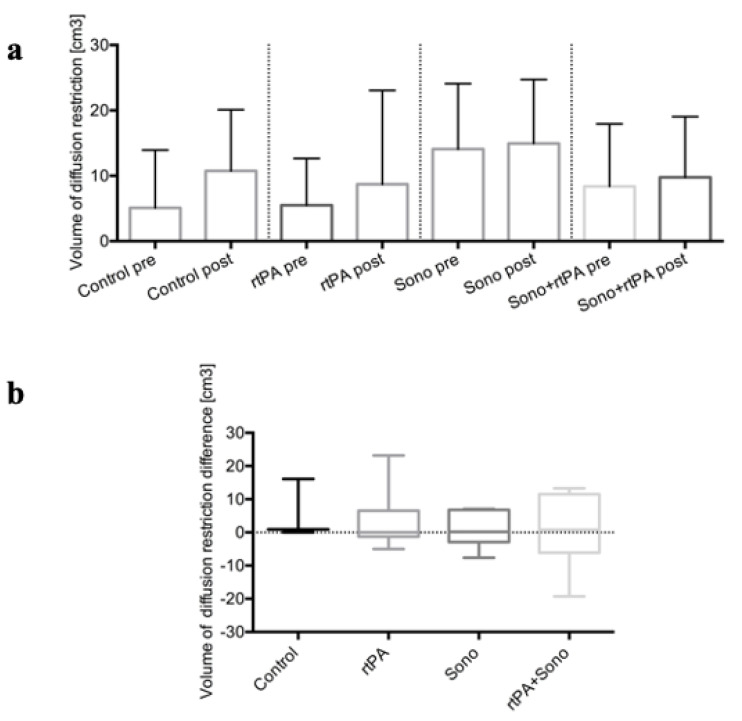
Cranial MRI (control group *n* = 3; treatment groups (2–4); each *n* = 6); (**a**) Volumes of diffusion restriction [cm3] (*y*-axis) of the different groups (1–4) (*x*-axis) before and after treatment are illustrated as a column bar graph plotting mean and SD. (**b**) Volumes of diffusion restriction difference [cm^3^] (*y*-axis) of the different groups (1–4) (*x*-axis) after treatment are depicted as box plots.

**Figure 4:**
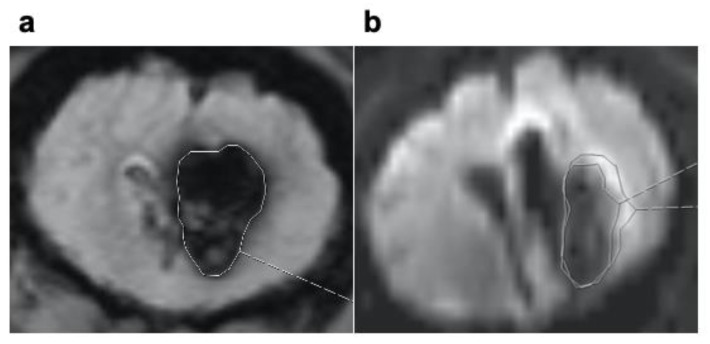
Cranial MRI a T2*- weighted image of (**a**) right frontal ICH white framed using the Sectra^®^ software (Linköping, Sweden). (**b**) DWI-image of a right frontal ICH. Diffusion restriction volume is white framed using the Sectra^®^ software.

Neither hyper- or hypoperfusion or intracranial vasoconstriction nor perihematomal edema were visible on cranial MRI. The volume of diffusion restriction (mean ± SD) was comparable in the control Group 1:5.1 ± 8.83 cm³; in Group 2: 5.51 ± 7.14 cm³; in Group 3: 14.1 ± 10 cm³, and in Group 4: 8.39 ± 9.53 cm³ after ICH placement. After treatment in the control group, the volume of diffusion restriction was 10.77 ± 9.33 cm³, in Group 2 it was 8.69 ± 14.38 cm, in Group 3 it was 14.95 ± 9.77 cm³, and in Group 4 it was 9.77 ± 9.27 cm³ (Figure 3a and Figure 4b). In all groups, there was a small increase in diffusion restriction (mean ± SD) after treatment, especially in the control Group; 5.67 ± 9.02 cm^3^ compared to Group 2 (rtPA): 3.18 ± 10.03 cm3, to Group 3 (ultrasound): 0.85 ± 5.48 cm3, and to Group 4 (rtPA + ultrasound): 0.82 ± 11.58 cm³ (Figure 3b). No correlation could not be found between initial ICH volume before treatment, and diffusion restriction volume before treatment (r = 0.275), and between ICH volume reduction after treatment, and diffusion restriction volume after treatment (r = −0.02).

There was one animal in Group 2 (rtPA), and another in Group 4 (rtPA and ultrasound) with a small perihematomal bleeding after ICH onset. These were both ≤0.6 cm³ and had a stable volume after treatment. 

### 3.4. HE-Staining: Microhemorrhages, Edema

**Figure 5:**
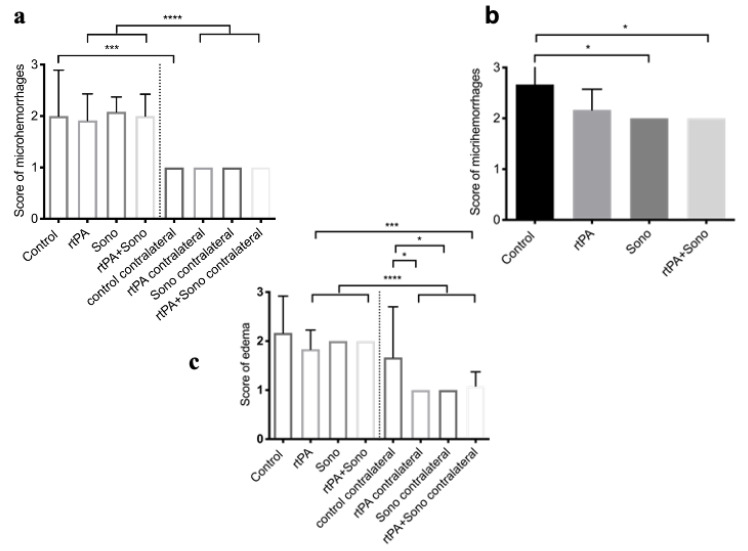
Microhemorrhages and edema using HE-staining (control Group *n* = 3; treatment groups (2–4); each *n* = 6). The means and SD of the score (1–3 points) of microhemorrhages or edema (*y*-axis) ipsilaterally and contralaterally to ICH or perihematomal (area 1) (*x*-axis) are plotted as a column bar graph. (**a**) **** marks the significant difference in the microhemorrhage-score of *p* < 0.0001 between the ipsilaterally and contralaterally brains of the ICH of the treatment groups (2–4). *** marks the significant difference of *p* = 0.0002 between the ipsi- and contralaterally brains of the control Group. (**b)** Perihematomal (area 1): * marks the significant difference of the control compared to Group 3 and 4 of *p* = 0.0258. (**c**) **** marks the significant difference of *p* < 0.0001 between the ipsilaterally and contralaterally brains of the treatment Groups (2–4). *** marks the significant difference of *p* = 0.0001 between the ipsilaterally Group 2 and the contralaterally Group 4. * marks the significant difference between the contralaterally control Group and the contralaterally Group 2 and 3 (*p* = 0.0148).

HE staining demonstrated perihematomal microhemorrhages (mean ± SD), which appeared primarily in the ipsilaterally hemispheres (areas 1 and 3), where the ICH was located (area 1), independent from treatment. The severity score of microhemorrhages of the ipsilateral side of the ICH of the control group was 2 ± 0.89 points, the score of Group 2 (rtPA) was 1.92 ± 0.52 points, the score of Group 3 (ultrasound) was 2.08 ± 0.29 points and the score of Group 4 (ultrasound + rtPA) was 2 ± 0.43 points. This was a significant occurrence compared to the contralaterally hemispheres, where no microhemorrhages were found. All groups achieved a score of 1 ± 0 points. The brains of the treatment groups (*p* < 0.0001) as well as the control Group (*p* = 0.0002) ipsilaterally to the ICH showed significantly more microhemorrhages compared to the contralaterally side (Figure 5a). In area 1, perihematomal, the control Group had a significantly higher microhemorrhage score of 2.67 ± 0.58 points compared to Group 3, who reached 2 ± 0 points, Group 4, also with 2 ± 0 points (*p* = 0.0258) and Group 2, who achieved 2.17 ± 0.41 points (Figure 5b). Comparing the anterior parts of the brains (areas 1, 2) with the posterior parts of the brains (areas 3, 4) the distribution of the microhemorrhages was equal between all groups. Anteriorly, the control group reached 1.83 ± 0.98 points and posteriorly 1.17 ± 0.41 points, Group 2 achieved 1.58 ± 0.67 points anteriorly and 1.33 ± 0.49 points posteriorly, Group 3 obtained 1.5 ± 0.52 points anteriorly and 1.58 ± 0.67 points posteriorly, and Group 4 achieved 1.58 ± 0.67 points anteriorly and 1.5 ± 0.67 points posteriorly.

**Figure 6:**
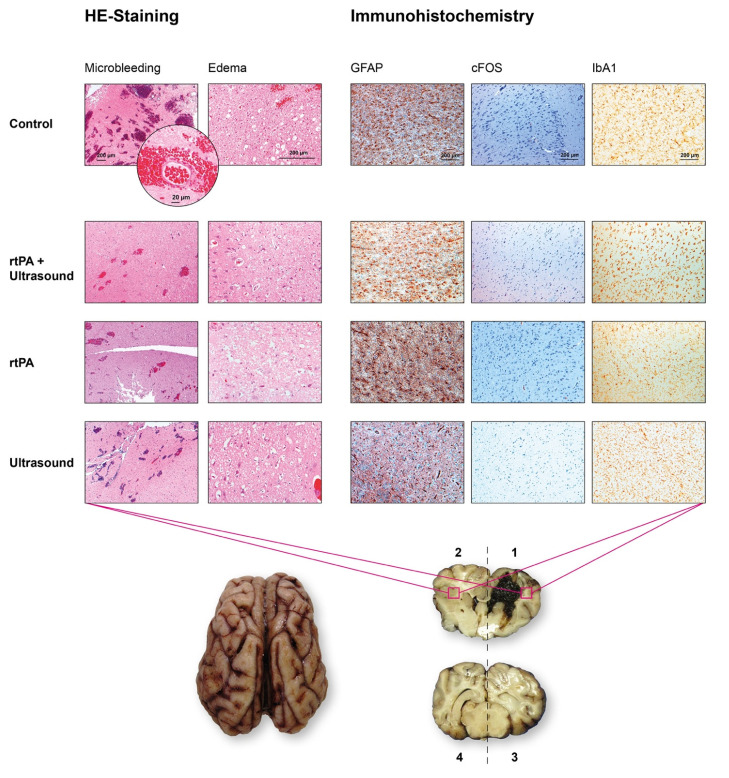
HE-staining and Immunohistochemistry. On the left side, there are representative HE-staining images. On the right side, representative immunohistochemistry images without significant differences are shown. All images were chosen from the brain area 1 (perihematomal, right frontal) and area 2 (left frontal, contralaterally), which are marked as red quadrats in the brain slice below the panels. The bar in each image represents 200 μm, except the round magnification of the first picture. Here, the bar represents 20 μm showing a typical perivascular microhemorrhage. Panel 1, row 1 shows microhemorrhages (3 points) and edema (2 points) using HE-staining, which is representative for area 1 of the control Group (Figure 5b). The HE-stain images of the other groups show a similar occurrence of microhemorrhages (each 2 points) and edema (each 2 points).

Compared to the distribution of microhemorrhages, there was more edema on the ipsilaterally side (areas 1, 3) than on the contralaterally side (areas 2, 4) (Figure 5c). Consequently, the score (mean ± SD) of the treatment groups was significantly higher ipsilaterally. Thus, Group 2 reached a score of 1.83 ± 0.39 points, while Group 3 (ultrasound) and Group 4 achieved a score of 2 ± 0 points. Compared to the contralaterally hemisphere, the score of edema for Group 2 and Group 3 was 1 ± 0 points, while Group 4 achieved 1.08 ± 0.29 points (*p* < 0.0001). This observation does not apply to the control group, which had higher scores in general and a similar score of 2.17 ± 0.75 points ipsilaterally and a score of 1.67 ± 1.03 points contralaterally, which was significantly higher compared to Groups 2 and 3 contralaterally (*p* = 0.0148) (Figure 5c and Figure 6).

Similar to the distribution of microhemorrhages, edema did not show a difference between the groups in the anterior (areas 1, 2) and posterior (areas 3, 4) parts of the brain. Anteriorly the control group reached 2 ± 0.89 points, and posteriorly 1.83 ± 0.98 points, Group 2 achieved 1.5 ± 0.52 points anteriorly and 1.42 ± 0.51 points posteriorly, Group 3 obtained 1.5 ± 0.52 points anteriorly and 1.5 ± 0.52 points posteriorly, and Group 4 similarly obtained 1.5 ± 0.52 points anteriorly and 1.58 ± 0.51 points posteriorly.

The perihematomal edema quantity of all groups was comparable: the control group reached a score of 2.33± 0.58 points, while Group 2 obtained a score of 1.83 ± 0.41 points and Groups 3 and 4 achieved a score of 2 ± 0 points.

### 3.5. Immunohistochemistry

The perihematomal (area 1) and contralaterally (area 2) immunohistochemistry of the brains of all groups appeared similar (Table 1 and Figure 6). Only GFAP was less dominant contralaterally in the brains, except for the control group (Table 1 and Table 2).

## 4. Discussion

To the best of our knowledge, this is the first time that the combined treatment of rtPA and ultrasound was performed in an in vivo ICH model. In the past, many trials have investigated the minimal invasive therapy of spontaneous ICH [8,9,24,30]. One important factor among ICH patients is a sufficient reduction in the ICH volume [8]. While in ischemic stroke, the transcranial use of ultrasound was the subject of many phase three clinical trials in the last twenty years [12,13,15,17,18,19,20,21,22,31,32], only one clinical study investigated the possibility of increasing rtPA fibrinolysis of ICH using intrahematomal ultrasound [11]. We previously described the optimal rtPA timing and dose in vitro [27] and analyzed the optimal ultrasound modalities in order to enhance rtPA clot lysis [28]. This was necessary to reduce the number of animals in this trial and to optimize conditions for further experimental and clinical trials on ICH in the future.

The established porcine model of ICH was chosen [29], including a necessary variation: instead of 1.7 mL autologous arterial blood, 5 mL was used in order to create similar ICH volumes compared to human ICH. This is important in order to improve evaluation of the effects of ultrasound with its individual lysis-radius [28]. In our model, it was possible to create reliable spherical porcine ICH volumes (Figure 2a and Figure 4). Vital signs were within the physiological range during experiments [33]. Interestingly, the ICP increased from 10 mm Hg on average to 80 mm Hg during the intracranial infusion of autologous blood, and it normalized over a period of 10–20 min after the procedure. This real-time ICP monitoring demonstrates the acute compensatory capacity of the brain, which could be one explanation why there are survivors during the acute phase despite large ICH volumes.

The results demonstrate that a significant hematoma reduction of approximately 60% is possible by a single application of the combined treatment of 1 mg rtPA and ultrasound, which is more than doubled compared to the use of rtPA (30%) and ultrasound (18%) alone. This is a promising volume reduction considering that up to 9 rtPA applications were performed in the MISTIE III protocol. These results are similar to our findings of our in vitro model of ICH, where the volume reduction is even better [28]. As previously shown in vitro, the superiority in clot thrombolysis of the combined treatment is based on an irreversible rarefication of the fine fibrin mesh of the clot [28]. It is thought that ultrasound leads to a temporary disruption of the cross-linked fibrin fibers and to a lowering of the clot density. This allows rtPA to enter the clot by acoustic streaming, so that the rtPA binding area is enlarged [26,34,35].

Another advantage of the combined treatment is that intracranial real-time imaging in b- and Doppler mode is possible in high quality [36], which could potentially minimize the risky transports of intensive care patients to the computer tomography (CT) scans.

There is some debate over ICH causes secondary neuronal damage and ischemia in several clinical and experimental trials [37,38,39,40]. The clear conception of a penumbra in ICH patients has not yet been established, perhaps due to heterogeneous observations and pathogenesis (ischemic, toxic, mechanical or mixed). Regarding this topic, in 36 patients, early CT images did not show an early mismatch between the perihematomal edema and the perihematomal hypoperfusion, so that tissue at risk of ischemia could not be detected [38]. Nevertheless, there are indications that in some patients, there is a diffusion restriction in DWI-MRI perihematomal within 6 h of symptom onset, and also a hypoperfusion of the hole hemisphere ipsilaterally to ICH [41]. In this trial, only one patient showed a combined perihematomal diffusion restriction and hypoperfusion, meaning that a perihematomal ischemia perhaps caused by mechanical vessel compression might be possible in some cases [41]. In a porcine model of ICH, after 6 h, a perihematomal ischemia was detected and its appearance was volume-dependent [42]. In addition, the perihematomal decrease of cerebral blood flow (CBF) is also known to be caused by toxic blood components of ICH (like matrix metalloproteinase, thrombin and iron) [43,44]. One PET study including 19 patients with ICH, found a reduced perihematomal CBF without increased oxygen extraction fraction, making local tissue damage by mechanical and toxic blood products more plausible than by ischemia [45]. There are hints that early perihematomal diffusion restriction seems to predict a worse clinical outcome [41,46].

The results from this study could not confirm a perihematomal penumbra at an ultra-early stage of ICH. Hypoperfusion was not apparent in PWI MRI, but 70% of all animals showed a rim of perihematomal diffusion restrictions, which did not correlate with the initial ICH volume. Additionally, the volume of diffusion restriction after therapy did not correlate with ICH volume reduction after therapy. This perihematomal diffusion restriction could be explained by relatively large ICH volumes in relation to the porcine brain. It is important to note that there was no significant increase in perihematomal diffusion restriction caused by therapy per se. These results have to be handled with care because it is possible that the MRI was performed too early (directly after ICH and after therapy, 3 h after ICH) to be able to detect perihematomal edema and significant changes in diffusion restriction volume. Whether the cause of these changes is ischemic, toxic, mechanical or mixed, however, remains unclear.

HE-stains revealed perivascular microhemorrhages (especially perihematomal), equally distributed in all treatment groups, and even more in the control group. This indicates a periprocedural phenomenon independent from ICH therapy. Early perihematomal edema was revealed by HE-stains, although edema was not visible on MRI yet. This is consistent with the findings of early perihematomal edema, despite an intact blood brain barrier (BBB) in animal models [29,47]. This observation was explained by increased interstitial serum protein accumulation, which was caused by increased hydrostatic pressure during hematoma formation, and clot retraction and by blood products [29,47].

The results from this study show a significantly enlarged edema of the control group (not only perihematomal but also contralateral), in contrast to the treatment groups. These results go hand in hand with the findings of the porcine animal model of ICH of Wagner et al., which revealed a reduction in perihematomal edema and prevention of vasogenic edema by early hematoma removal, using rtPA-fibrinolysis [48].

Secondary brain injury (SBI) is a major influencing factor causing functional impairment. It results from the ICH’s hemoglobin breakdown products, which initiate an inflammation process, causing oxidative stress, neurotoxicity, apoptosis, followed by the disruption of the blood brain barrier and edema [49,50]. To identify SBI, immunohistochemistry was performed. The control group and the different treatment groups did not distinguish between neuronal activation using c-Fos stain, an unspecific marker for neuronal stress [49], and the microglial activation using Iba1 immunohistochemistry [50]. Microglia act as key immune cells and are the first non-neuronal cells responding to brain injury by the production of cytokines, chemokines, proteases, prostaglandins, and various other proinflammatory products. These cells initiate oxidative and cytotoxic cascades, depending on their occurrence and microglial polarization (M1 or M2). Though the detection of these first-line immune cells was chosen, the time from therapy to fixation of the brains might have been too short to detect differences in this Iba1-marked, immune cell activation, which could initiate secondary brain injury or brain repair after ICH [50].

In contrast, similar to the occurrence of edema, there was a larger area of astroglial marker GFAP of the control group (contralaterally of the ICH) compared to the treatment groups. This could indicate a stronger early glial activation of the untreated group. The finding is supported by the fact that the GFAP serum concentration is an early biomarker of patients with ICH. This can help to distinguish these patients from those with ischemia in the early preclinical phase [51,52,53,54]. This early increase in GFAP is an expression of an increased blood brain barrier disruption, and astroglial destruction in ICH patients, compared to ischemic stroke patients [54]. GFAP levels seem to correlate with increasing ICH volume and worse clinical outcomes [54]. This could also be a surrogate marker of ICH development and therapy in the future.

In conclusion, in the ultra-early phase of ICH, neither MRI nor HE staining nor immunohistochemistry showed adverse side effects of the more effective combined treatment of rtPA and ultrasound. Side effects are known to exist in some ultrasound circumstances and modalities, which were excluded using an animal model [13,14,17,18,20,22].

The extrapolation of these ultra-early findings from animals to human patients has to be done with care. Still, there are clear limitations to our study. This animal model, where blood is injected into the brain, does not completely mirror the pathophysiological reality of a spontaneous ICH and its microvascular milieu in humans, which suffer from spontaneous vessel rupture. The proportions of the human brain and skull are comparable to pigs but there are still differences in the anatomy and in the dimensions of the skull and the brain. A considerable portion of patients suffering ICH is also affected by comorbidities and consumption of blood diluting drugs, which can influence the therapeutic results. Additionally, the total number of animals is limited. In this study, MRI, histopathology and immunohistochemistry of the brains after a longer period are missing; these would be needed to evaluate the secondary injury. Further experiments with a higher number of animals, a longer observation time including a neurological assessment are required to evaluate the secondary complex inflammatory brain injury, recovery mechanisms and clinical outcome. There is no doubt that the focus in ICH research, the clinical outcome, is a complex and multifactorial product of patient’s parameters, such as ICH size, location, size reduction, toxic blood breakdown products, inflammation and oxidative stress resulting in secondary brain injury. Nevertheless, there is limited evidence that the surgical elimination of these toxic blood degradation products in combination with volume reduction can be beneficial and will be an important part of the treatment of ICH in the future.

## 5. Conclusions

In an established large-animal model of ICH, we were able to demonstrate the clear effectiveness of minimally invasive, catheter-based rtPA fibrinolysis and ultrasound thrombolysis by an intrahematomal ultrasound catheter. The combined intrahematomal application of 1 mg rtPA and high-frequency ultrasound using 10 MHz for one hour is a significantly more effective hematoma-reducing therapy compared to the use of ultrasound or rtPA only and compared to the control group. Biological effects on ICH and the surrounding brain tissue could be evaluated in vivo at an ultra-early stage without detecting side effects of the different treatments compared to the control group.

## 6. Patents

This publication is related to a patent: EP 3 228 266 A1: A device for ultrasonic-accelerated hematoma lysis or thrombolysis of intracerebral or intraventricular hemorrhages or hematomas.

## Figures and Tables

**Table 1:** Score of immunohistochemistry perihematomal (area 1) and contralaterally (area 2) to the ICH (control Group *n* = 3; treatment groups (2–4); each *n* = 6).

	Control	rtPA	Ultrasound	rtPA + Ultrasound
	(Area 1)	(Area 1)	(Area 1)	(Area 1)
GFAP	2.67 ± 0.58	2.33 ± 0.52	2.5 ± 0.55	2.33 ± 0.52
cFos	1.33 ± 0.58	1.17 ± 0.41	1.67 ± 0.82	1.83 ± 0.75
Iba1	2 ± 1.00	2.5 ± 0.55	2.5 ± 0.84	2.5 ± 0.84
	Control contralaterally	rtPA contralaterally	Ultrasound contralaterally	rtPA + Ultrasound contralaterally
	(area 2)	(area 2)	(area 2)	(area 2)
GFAP	2 ± 1	1.33 ± 0.52	1.33 ± 0.52	1 ± 0
cFos	1.67 ± 0.58	1.33 ± 0.82	1.67 ± 1.03	2 ± 0.89
Iba1	1.67 ± 0.58	2 ± 0.63	2 ± 0.63	2 ± 0.63

**Table 2:** *p* values of the comparison between GFAP immunohistochemistry perihematomal (area 1) and contralaterally (area 2) to the ICH.

rtPA (area 1) vs. Sono contralaterally (area 2)	0.0444	Sono (area 1) vs. rtPA + Sono contralaterally (area 2)	0.0005
rtPA (area 1) vs. rtPA + Sono contralaterally (area 2)	0.0025	rtPA + Sono (area 1) vs. rtPA contralaterally (area 2)	0.0444
Sono (area 1) vs. rtPA contralaterally (area 2)	0.0112	rtPA + Sono (area 1) vs. Sono contralaterally (area 2)	0.0444
Sono (area 1) vs. Sono contralaterally (area 2)	0.0112	rtPA + Sono (area 1) vs. rtPA + Sono contralaterally (area 2)	0.0025

## Data Availability

All datasets analyzed in this study are available from the corresponding author on reasonable request. Results of this work are part of the ongoing doctoral thesis of C.S.
